# Antidiabetic Potential of *Abelmoschus manihot* Flower Extract: In Vitro and Intracellular Studies

**DOI:** 10.3390/medicina60081211

**Published:** 2024-07-26

**Authors:** Shih-Wei Wang, Thung-Lip Lee, Tzu-Hsien Chang, Ya-Ling Chen, Hsin-Ya Houng, Natasha Chang, Sabrina Chang, Chi-Chang Chang, Jer-Yiing Houng

**Affiliations:** 1 School of Medicine, College of Medicine, I-Shou University, Kaohsiung 82445, Taiwan; ed101397@edah.org.tw; 2 Division of Allergy, Immunology, and Rheumatology, Department of Internal Medicine, E-Da Hospital, I-Shou University, Kaohsiung 82445, Taiwan; 3 Division of Allergy, Immunology, and Rheumatology, Department of Internal Medicine, E-Da Dachang Hospital, I-Shou University, Kaohsiung 80706, Taiwan; 4 School of Medicine for International Students, College of Medicine, I-Shou University, Kaohsiung 82445, Taiwan; ed102601@edah.org.tw; 5 Division of Cardiology, Department of Internal Medicine, E-Da Hospital, I-Shou University, Kaohsiung 82445, Taiwan; 6 Department of Obstetrics & Gynecology, E-Da Hospital, I-Shou University, Kaohsiung 82445, Taiwan; ed112550@edah.org.tw (T.-H.C.); ed109776@edah.org.tw (Y.-L.C.); nhy8381@gmail.com (H.-Y.H.); 7 Department of Obstetrics & Gynecology, E-Da Dachang Hospital, I-Shou University, Kaohsiung 80706, Taiwan; 8 Sayles Hill Campus Center, Carleton College, Northfield, MN 55057, USA; Natashachang1010@gmail.com (N.C.); Sabrinachang1010@gmail.com (S.C.); 9 Department of Nutrition, I-Shou University, Kaohsiung 82445, Taiwan; 10 Department of Chemical Engineering, I-Shou University, Kaohsiung 82445, Taiwan

**Keywords:** *Abelmoschus manihot* (L.) Medic flower, advanced glycation end-products, carbohydrate-digesting enzyme, pancreatic β-cells, cell viability, insulin secretion

## Abstract

*Abelmoschus manihot* (L.) Medic flower (AMf) exhibits both nutritional value and bioactivities such as antioxidative, anti-inflammatory, neuroprotective, cardioprotective, and hepatoprotective effects. The aim of this investigation was to examine the potential impact of three different solvent extracts of AMf: supercritical CO_2_ extraction extract, water extract, and ethanol extract (AME), on management of diabetes. All three extracts demonstrated significant inhibitory effects on α-glucosidase (IC_50_ = 157–261 μg/mL) and lipase (IC_50_ = 401–577 μg/mL) activities while enhancing the α-amylase activity (32.4–41.8 folds at 200 μg/mL). Moreover, all three extracts exhibited notable inhibition of the formation of advanced glycation end-products, including the Amadori products (inhibition rates = 15.7–36.6%) and the dicarbonyl compounds (inhibition rates = 18.6–28.3%). Among the three extracts, AME exhibited the most pronounced inhibitory effect. AME displayed substantial in vitro and intracellular antioxidative activity, and effectively reduced ROS production (135% at 500 μg/mL) in β-cells under hyperglycemic (HG) conditions. AME also enhanced the activity and gene expression of antioxidant enzymes, which were markedly decreased in the HG-induced β-cells. Furthermore, AME protected β-cell viability and maintained normal insulin secretion under HG conditions, likely due to its ability to reduce oxidative stress within β-cells. This study demonstrated the potential of AME in preventing and managing diabetes and its associated complications. Further in vivo research is necessary to thoroughly elucidate the preventive effects and their underlying mechanisms.

## 1. Introduction

Diabetes mellitus is a chronic metabolic disorder characterized by prolonged elevation of blood sugar levels. Type 1 diabetes, a congenital insulin deficiency, is caused by the destruction of pancreatic β-cells which are responsible for insulin production. By comparison, type 2 diabetes is caused by the inability of pancreatic β-cells to adequately secrete insulin or the development of insulin resistance. Under normal circumstances, pancreatic β-cells, which exhibit high sensitivity to blood glucose levels, regulate blood sugar homeostasis through insulin secretion. However, a prolonged exposure to hyperglycemia can have deleterious effects on β-cells, a phenomenon known as “glucotoxicity”. These effects reduce responsiveness to glucose stimulation, decrease insulin secretion, impair reactions to insulin-stimulating factors, and can even trigger the immune system to mistakenly target and destroy β-cells, causing insulin deficiency. Progressively, these adverse effects cause a gradual decline in the quantity, integrity, and functionality of β-cells. Thus, the condition of β-cells plays as a pivotal factor in the progression of type 2 diabetes [1,2].

The carbohydrate-digesting enzyme inhibitor has been shown to be an effective therapeutic strategy for treatment of type 2 diabetes. It inhibits the activity of pancreatic α-amylase and intestinal α-glucosidase to reduce starch hydrolysis and intestinal digestion and absorption of sugar, thereby controlling postprandial blood sugar levels [3,4].

Diabetes can also have adverse effects on lipid metabolism. In patients with type 2 diabetes, insulin resistance can lead to abnormalities in lipid metabolism, such as an increased level of triglycerides and low-density lipoprotein cholesterol levels, and a decreased level of high-density lipoprotein cholesterol. Dysfunctional lipid metabolism often poses a cardiovascular disease risk. Pancreatic lipase, an enzyme responsible for breaking down dietary lipids into fatty acids and glycerol, is critical in this metabolic process. Therefore, inhibiting the lipase activity to reduce the absorption of dietary lipids may be a good strategy to control the progression of diabetes [4].

Glycation involves a non-enzymatic process characterized by the interaction between aldehyde groups in sugars and amino groups in proteins, nucleic acids, and lipids. This interaction at the start forms a reversible Schiff base, which is then transformed into an Amadori product through rearrangement. Subsequently, these initial glycation products undergo further chemical transformations such as dehydration, oxidation, and cyclization, with dicarbonyl compounds, such as glyoxal, methylglyoxal, and glycolaldehyde. These changes eventually result in a permanent alteration of proteins and generation of advanced glycation end-products (AGEs) [5]. The production and accumulation of these AGEs, which can contribute to various pathological processes, are accelerated by elevated blood sugar levels, oxidative stress, and inflammatory conditions. Conversely, AGEs can trigger oxidative stress and inflammation by interacting with AGE receptors in various cells. Consequently, the excessive accumulation of AGEs can readily lead to the onset and progression of diverse metabolic disorders and the aging process, and give rise to diabetes-related complications, such as peripheral neuropathy, nephropathy, cardiovascular complications, ocular disorders, and atherosclerosis. Hence, inhibition of the glycation cascade has emerged as a promising therapeutic strategy to prevent or treat diabetes and its associated pathogenic complications [6,7,8].

Evidence has also shown that a hyperglycemic (HG) environment could increase the production of AGEs and reactive oxygen species (ROS) by mitochondria. This dual effect can cause substantial oxidative stress on cells and induce dysfunction and apoptosis in β-cells, tissue damage, insulin resistance, and aging [9,10]. Additionally, glucotoxicity can lead to reduced antioxidant enzyme activity [1]. These deleterious effects of glucotoxicity will increase the incidence of various diabetic complications and exacerbate the difficulty of blood sugar control [11,12]. Therefore, it is crucial to identify a strategy that can scavenge excess free radicals to alleviate the hyperglycemia-induced oxidative stress and protect pancreatic β-cells, thereby reducing the risk of diabetes-related pathological damage [1].

The RIN-m5f cell line, derived from a rat insulinoma, exhibits remarkable stability and effectively simulates pancreatic β-cell function. These cells’ insulin-secreting capability renders them suitable for in vitro studies focusing on insulin secretion. These studies are instrumental in elucidating the molecular mechanisms underlying diabetes and in fostering the development of innovative therapeutic strategies. Therefore, RIN-m5f cells have become a commonly used model for investigating the roles and mechanisms of insulin secretion in diabetes research [13,14].

Aibika (*Abelmoschus manihot* (L.) Medic) is a globally distributed flowering plant of the Malvaceae family. Traditionally, the *A. manihot* flower (AMf) has been used in dishes to add flavor, texture, and nutritional value, and has also been used to make scented tea products. AMf has also been utilized for topical or oral administration to reduce swelling, detoxify, promote blood circulation and hemostasis, and facilitate diuresis and urination. Additionally, it has been used in the treatment of burns, malignant skin ulcers, and cellulitis. Moreover, AMf has demonstrated efficacy against various conditions including cerebral infarction, osteoporosis, gastrointestinal disturbances, menstrual bleeding management, alleviation of labor pains, promotion of lactation, and enhancement of sexual arousal and reproductive capacity [15,16]. Extracts from AMf, which are rich in bioactive constituents, exhibit diverse bioactivities, including antioxidant, anti-inflammatory, anti-lipogenic, anti-convulsant, anti-depressant, anti-viral, analgesic, immunomodulatory, neuroprotective, cardioprotective, and hepatoprotective properties. In China, extracts from AMf are employed in clinical treatments for diabetic nephropathy and chronic glomerulonephritis [17,18]. However, despite its extensive applications, the mechanism underlying its impact on the occurrence and progression of diabetes has not been reported in the literature. The aim of this investigation was to assess the potential of AMf in the prevention and treatment of diabetes, encompassing its in vitro bioactivity and its potential to protect pancreatic β-cells from damage induced by elevated glucose levels.

## 2. Materials and Methods

### 2.1. AMf Extraction Procedures

The dried AMf was purchased from Bozhou (Anhui, China). AMf was pulverized into a fine powder. For preparation of the AMf water extract (AMW), 1.0 kg of dried flower powder was added into 6 L of water and heated at 90 °C for 3 h. After cooling to room temperature, the extract was filtered and water in the filtrate was removed by an evaporator (Panchum Scientific, Kaohsiung, Taiwan). The resulting residue was freeze-dried to obtain the AMW extract, yielding an extraction rate of 16.4%.

The AMf ethanol extract (AME) was prepared by extracting 2.6 kg of AMf powder three times using 3 × 16 L of 95% ethanol, with a duration of 24 h per extraction. After filtering the three extraction solutions, the alcohol was evaporated and the residue freeze-dried to obtain the dried AME extract, achieving an extraction rate of 25.2%.

The CO_2_ supercritical fluid extract (AMS) was produced using a supercritical fluid extractor (Natex, Ternitz, Austria). The AMf powder of 1.0 kg was introduced into the extraction chamber, where the temperature was gradually increased to 40 °C. Subsequently, the pressure was incrementally raised to 150 bar within 20 min, then increased to 250 bar over the next 20 min, further to 300 bar within the subsequent 10 min, and finally to 350 bar within another 10 min, where it remained for 2 h. After pressure was released, the residue underwent freeze-drying to yield the AMS extract, with an extraction yield of 1.0%.

The measurement of the concentrations of five flavonoid compounds in the extracts utilizing HPLC and the analysis of total polyphenol content (TPC) and total flavonoid content (TFC) were all conducted in accordance with the conditions outlined in the study by Chang et al. [16]. Before usage, the three dry extracts were kept in a refrigerator at −20 °C.

### 2.2. Enzyme Activity Assays

The following enzyme activity assays were based on the methods described by Palanisamy et al. [19] and McDougall et al. [20] with some modifications.

(A) α-Glucosidase

Quantities of 50 μL of extract samples, 25 μL glutathione solution, and 25 μL α-glucosidase (*Saccharomyces cerevisiae*, Sigma-Aldrich Chemicals, St. Louis, MO, USA) were dissolved in phosphate buffer (pH 6.9). The reaction was started by adding 20 μL of 5 mM p-nitrophenyl-α-D-glucopyranoside. After 15 min incubation at 37 °C, the reaction was stopped by adding 80 µL of 0.2 M sodium carbonate solution. The absorbance of the reaction mixture was detected at 405 nm by a Bio-Rad ELISA reader (Model 550, Hercules, CA, USA). The inhibition rate of enzyme activity was then calculated as:

Inhibition % = [(A_C_ − A_S_ + A_S__−__B_)/(A_C_ − A_B_)] × 100% (1)

In this equation, A_C_ represents the absorbance observed when the carrier phosphate buffer instead of the sample solution was used in testing. A_S_ denotes the absorbance of the sample solution itself. A_S−B_ indicates the absorbance when sodium carbonate was added before the reaction and then followed the same steps. A_B_ is the absorbance recorded under conditions where the sample solution was replaced by phosphate buffer, and sodium carbonate solution was added before the reaction, with the subsequent procedure maintained.

(B) α-Amylase

A quantity of 80 µL of the extract solution was mixed with 40 µL of α-amylase (porcine pancreas type IV-B, Sigma-Aldrich) solution and preheated at 37 °C. Next, 40 µL of 1% soluble potato starch solution was introduced and the reaction was allowed to proceed at 37 °C. After 10 min of incubation, 80 µL of 3,5-dinitrosalicylic acid solution was added and further incubated at 95 °C for 10 min. After cooling, the absorbance (at 540 nm) of the mixture was measured and the enzyme activity inhibition rate was computed using Equation (1).

(C) Lipase

The substrate solution consisted of 1.6 mM p-nitrophenyl laurate supplemented with 1% Triton X-100. Prior to the assay, 50 μL of the extract solution and 50 μL of lipase (porcine pancrease Type II, Sigma-Aldrich) solution were mixed and preheated at 37 °C. Then, the substrate solution was added to initiate the reaction, which proceeded at 37 °C for 30 min. The reaction was terminated by heating the reaction mixture at 85 °C for 5 min. After centrifugation at 2500× *g* for 5 min, the absorbance of the supernatant was detected at 405 nm, and the enzyme activity inhibition rate was calculated using Equation (1).

### 2.3. Effect on AGEs Formation

The analysis was performed according to the method of Manaharan et al. [21] with some modifications. The bovine serum albumin (BSA)−glucose glycation system was utilized to mimic the generation of AGEs. The inhibitory impact on the generation of Amadori products and dicarbonyl compounds during the glycosylation process was assessed using the nitroblue tetrazolium (NBT) reduction and the Girard-T assay, respectively. The experimental procedure included mixing 8 mL, 10 mg/mL BSA solution, 1.6 mL, 1 M glucose solution, and 0.4 mL extract solution. The mixture was reacted at 80 °C for 7 days, and aliquots of samples were removed on different days for assays of glycated material solutions.

For NBT reductive assay, a mixture comprising 0.5 mL of glycated material solution and 2.0 mL, 0.3 mM NBT reagent was mixed and then incubated at room temperature for 15 min. Subsequently, the absorbance of the solution was measured at 530 nm.

For Girard-T assay, a volume of 0.4 mL of glycated material solution was mixed thoroughly with 0.2 mL of Girard’s reagent T solution (0.5 M) and 3.4 mL of sodium formate (0.5 M). The mixture was then incubated at room temperature for 1 h, and the absorbance was measured at 295 nm. The content of dicarbonyl compounds in the sample was estimated using a calibration line with glyoxal (Sigma-Aldrich) serving as the standard.

### 2.4. Cultivation of β-Cells

The RIN-m5F rat pancreatic β-cell line (BCRC 60410) was acquired from the Bioresource Collection and Research Center (Hsinchu, Taiwan). This cell line was cultured in RPMI 1640 medium supplemented with 100 U/mL penicillin, 100 μg/mL streptomycin, 2 mM L-glutamine, and 10% Fetal bovine serum (FBS, Gibco Co., Grand Island, NY, USA). The incubator conditions were set at 37 °C, 5% CO_2_, and 95% air.

The experimental conditions were designed based on the work of Rugină et al. [22]. In general cultivation, RIN-m5F cells were seeded at 5 × 10^4^ cells/well in a 96-well plate and cultured for 24 h. To examine the survival rate of RIN-m5F cells, extract samples dissolved in dimethyl sulfoxide (DMSO), with or without glucose, were added to the cells, ensuring a final DMSO concentration of less than 0.1% to mitigate any impact on cell growth. Following a 24 h incubation, the culture medium was replaced with a medium containing MTT assay kit (Sigma-Aldrich). After a 4 h incubation, the medium was replaced by DMSO solution, and vigorously shaken to dissolve all crystals, and the survival rate of RIN-m5F cells was determined at 570 nm.

### 2.5. Scavenging Activity on DPPH Radicals

The experimental method was designed based on the study of Tsai et al. [23]. A volume of 100 µL of sample solution was mixed with 25 µL of 0.5 mM 1,1-diphenyl-2-picrylhydrazyl (DPPH) solution, and allowed to react in darkness for 30 min. Following completion of the reaction, the absorbance was measured at 517 nm. In control experiments, the extract sample was replaced with ethanol, while in blank experiments, DPPH in the reaction solution was replaced with ethanol. The DPPH radical scavenging rates were subsequently determined as:

DPPH scavenging rate (%) = [1 − (A_S_ − A_B_)/A_C_] × 100% (2)

where A_S_, A_B_, and A_C_ are the absorbance of the sample, the blank, and the control solutions, respectively.

### 2.6. Scavenging Activity on ABTS Radicals

The analytical method followed the study by Patra et al. [24] with some alterations. A 7.4 mM solution of 2,2′-azino-bis(3-ethylbenzothiazoline-6-sulfonic acid) (ABTS) was mixed with a 2.6 mM potassium persulfate solution at a 1:1 volume ratio and kept in the dark at room temperature for 12–16 h. After adding 20 µL of the sample solution to 180 µL of this mixture, the solution was incubated at 37 °C in the dark for 2 h. The absorbance of the reaction solution was then detected at 735 nm. The control was measured using ethanol instead of the sample solution. The ABTS radical scavenging rates were determined as follows:

ABTS•^+^ scavenging activity (%) = (A_C_ − A_S_)/A_C_ × 100% (3)

### 2.7. Intracellular ROS Level Analysis

The analysis method was based on the study conducted by Chen et al. [25], with minor modifications. RIN-m5f cells were seeded at 1 × 10^5^ cells/well and cultured for 24 h. Afterward, the culture medium was replaced with fresh medium containing extract solutions of varying concentrations (0, 125, 250, 500 μg/mL) and specified concentrations of D-glucose (50 and 100 mM). Following an additional 24 h cultivation, the cells were washed with phosphate-buffered saline (PBS) and subjected to the OxiSelect^TM^ ROS Assay Kit (Cell Biolabs, San Diego, CA, USA). After incubation in darkness for 1 h, the medium was substituted with PBS, and the intracellular ROS levels were assessed using a microplate reader (Synergy^TM^ 2, BioTek, Winooski, VT, USA) equipped with fluorescence detection. Excitation and emission wavelengths were set at 502 nm and 524 nm, respectively, for this assessment.

### 2.8. Antioxidant Enzymes Activity Assay

Cell extracts were obtained by adding the cell lysate to the prepared RIN-m5f cells for protein extraction. Activity assays for catalase, glutathione peroxidase (GPx), and superoxide dismutase (SOD) were performed using Sigma-Aldrich kits. Quantification of protein content in the samples was conducted using the BCA Protein Assay Kit (Sigma-Aldrich).

### 2.9. Gene Expression Analysis

Cells were harvested and subjected to RNA isolation using the Qiagen RNeasy Kit (Qiagen, Venlo, The Netherlands). The isolated RNA underwent reverse transcription to generate cDNA with the Magic RT cDNA Synthesis Kit (Bio-Genesis, Taipei, Taiwan). Following this, the resultant cDNA was amplified using the IQ2 SYBR Green Fast qPCR Synthesis Master Mix LOW ROX Kit (Bio-Genesis) on a Real-Time PCR Instrument (7500 Fast Dx, Applied Biosystems, Foster City, CA, USA). The primers utilized in RT-qPCR gene amplification operations are listed in Table 1. The experimental protocol comprised an initial reaction stage at 50 °C for 2 min, followed by a subsequent stage at 95 °C for 10 min. This was succeeded by a third stage reaction at 95 °C for 15 sec, and a subsequent reaction at 60 °C for 1 min. The entire process encompassed a total of 40 cycles.

### 2.10. Insulin Secretion Analysis

The medium in the treated RIN-m5f cell culture dish was replaced with Krebs-Ringer buffer supplemented with 2% FBS and 2.5 mM glucose. The culture was then incubated at 37 °C for 30 min, followed by an insulin secretion assay using the Rat/Mouse Insulin ELISA Kit (Sigma-Aldrich).

### 2.11. Statistical Analysis

Each experiment was replicated independently three to five times. The results are presented as means ± standard deviation. The significance of differences was determined through one-way analysis of variance (ANOVA), with significance levels indicated as * *p* < 0.05, ** *p* < 0.01, *** *p* < 0.001. Statistical difference analyses were carried out using SPSS 25.0 software (IBM Inc., Armonk, NY), while other statistical computations were performed using Excel software (Office 2019, Microsoft Software Inc., Redmond, WA, USA).

## 3. Results

### 3.1. Inhibitory Effects on Related Enzymes

**Figure 1:**
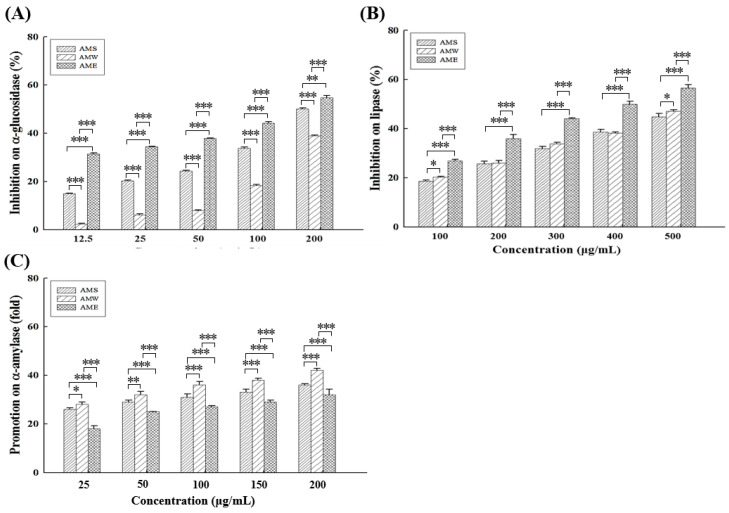
Inhibitory activities of the three AMf extracts against α-glucosidase (**A**), lipase (**B**), and α-amylase (**C**), at different concentrations. Each set of data was derived from three separate replicate experiments. Statistical differences were determined using one-way ANOVA test. Levels of significance are indicated as * *p* < 0.05, ** *p* < 0.01, and *** *p* < 0.001.

All three different AMf extracts exhibited dose-dependent inhibitory effects on α-glucoamylase (Figure 1A) and lipase (Figure 1B) activities. Among them, AME (IC_50_ = 157.4 µg/mL) exhibited the highest α-glucoamylase inhibitory activity, followed by AMS (IC_50_ = 196.0 µg/mL) and AMW (IC_50_ = 260.7 µg/mL). At a concentration of 200 µg/mL, the inhibition rates of AME, AMS, and AMW on α-glucoamylase activity were 54.7%, 50.1%, and 38.8%, respectively, while the positive control acarbose exhibited an 80.4% inhibition rate. In terms of lipase inhibitory activity, the order was AME (IC_50_ = 400.6 µg/mL) > AMW (IC_50_ = 558.3 µg/mL) > AMS (IC_50_ = 576.7 µg/mL), at 500 µg/mL, and the rates were 56.5%, 47.0%, and 44.8%, respectively.

Different from the inhibitory effects on α-glucoamylase and lipase, all three extracts displayed a dose-dependent promotion rate on α-amylase activity (Figure 1C), suggesting a potential facilitation of starch digestion in the intestine. The order of the improvement multiplier was AMW > AMS > AME. At a concentration of 200 µg/mL, AMW, AMS, and AME increased α-amylase activity by 41.8, 36.0, and 32.4 fold, respectively.

### 3.2. Inhibitory Effects on AGE Formation

**Figure 2:**
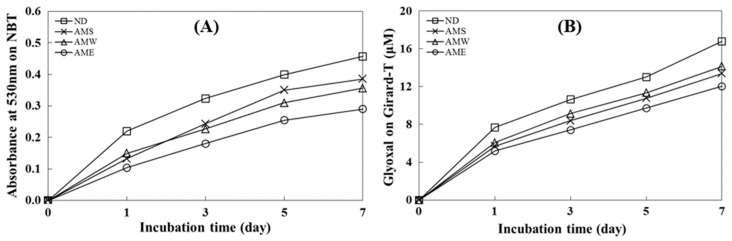
Effects of AMf extracts on the formation of AGEs. (**A**) Analysis of Amadori product formation by measuring the reduction in NBT; (**B**) assessment of dicarbonyl compound production using the Girard-T assay. The concentration of the extract tested was 1 mg/mL.

The data presented in Figure 2A illustrate an incubation time-dependent increase in glycated BSA formation as the absorbance value at 530 nm increased. This finding shows the inhibitory ability of the three AMf extracts on the formation of Amadori products. The order of suppression was AME > AMW > AMS. On day 7, the inhibition rates for these extracts were 36.6%, 22.0%, and 15.7%, respectively.

Figure 2B depicts an incubation time-dependent increase in the quantity of dicarbonyl compounds generated through glycation. All three AMf extracts exhibited the capacity to suppress the formation of dicarbonyl compounds, with the order of inhibitory activity being AME > AMS > AMW. On day 7, the inhibition rates for these extracts were 28.3%, 20.2%, and 18.6%, respectively.

### 3.3. Effects on Proliferation of HG-Induced β-Cells

**Figure 3:**
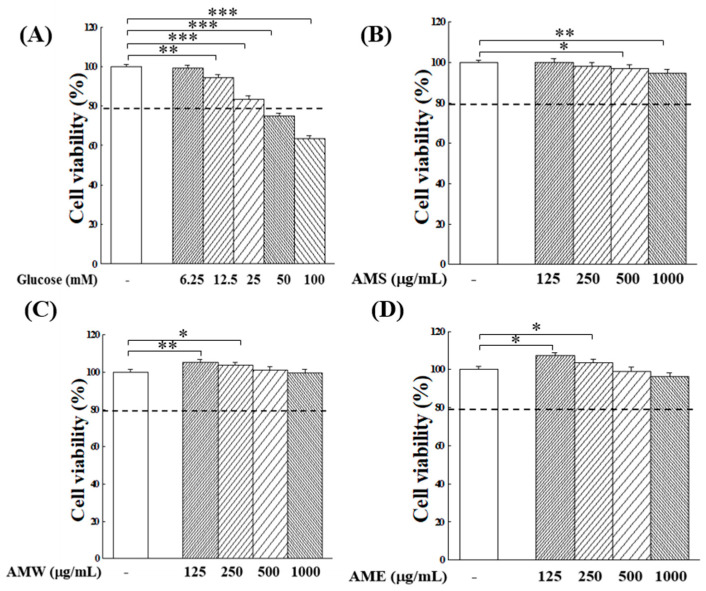
Effects of varying concentrations of glucose (**A**), AMS (**B**), AMW (**C**), and AME (**D**) on the proliferation of RIN-m5f β-cells. The control group consisted of untreated RIN-m5f cells, with the cell count after incubation set at 100%. The dashed line is the auxiliary line for judgment and represents 80% survival rate. Data were collected from five independent experiments. Statistical differences were determined using one-way ANOVA test. Levels of significance are indicated as * *p* < 0.05, ** *p* < 0.01, *** *p* < 0.001.

The initial exploration focused on examining how varying glucose concentrations affect the proliferation of RIN-m5f β-cells. The experimental findings presented in Figure 3A indicate that increasing glucose concentration exerts a substantial inhibition on β-cell growth. Specifically, at glucose concentrations in medium at 50 or 100 mM, cell viability decreased to 75% and 61% of the control group, respectively. This underscores the cytotoxic impact of elevated glucose levels on RIN-m5f cells. In the following studies, the application of AMf extracts on the proliferation of RIN-m5f β-cells under highly stringent conditions of 50 or 100 mM glucose concentrations was assessed to better elucidate the potential protective effects of AMf extracts.

Next, the impact of varying concentrations of AMf extracts on the proliferation of RIN-m5f cells was investigated. The results presented in Figure 3B–D reveal that, at low concentrations (<250 µg/mL), both AMW and AME stimulated the growth of β-cells. However, at a high concentration of 1000 µg/mL, they displayed a marginal inhibitory effect on cell proliferation. In spite of that, within the tested concentration range, the survival rates of RIN-m5f cells remained above 95% after 24 h of culture.

**Figure 4:**
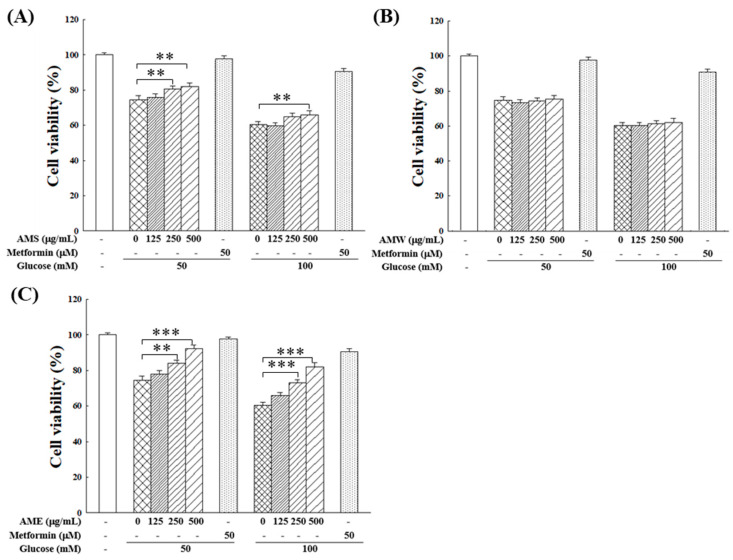
Effects of varying concentrations of AMS (**A**), AMW (**B**), and AME (**C**) on the proliferation of RIN-m5f β-cells under HG stimulation. The control group consisted of untreated RIN-m5f cells, with the cell count after incubation set at 100%. Data were collected from five independent experiments. Statistical differences were determined using one-way ANOVA test. Levels of significance are indicated as ** *p* < 0.01, *** *p* < 0.001. Metformin of 50 µM was used as the positive control.

Subsequently, the influences of AMf extracts on RIN-m5f cell proliferation under the HG condition were examined. Figure 4 illustrates that within the tested range of 0–500 µg/mL, AMW showed no apparent effect, while AMS exhibited a marginal improvement in RIN-m5f cell proliferation under HG conditions. On the other hand, AME displayed a notable protective effect, which was intensified with increasing doses. At a concentration of 500 µg/mL, AME raised the cell survival rate to 92.1% (50 mM glucose) and 82.2% (100 mM glucose). This protective effect on cell proliferation closely resembled that of the positive control, 50 µM metformin, which is a clinical medicine for type 2 diabetes.

### 3.4. In Vitro and Intracellular Antioxidant Effects

**Figure 5:**
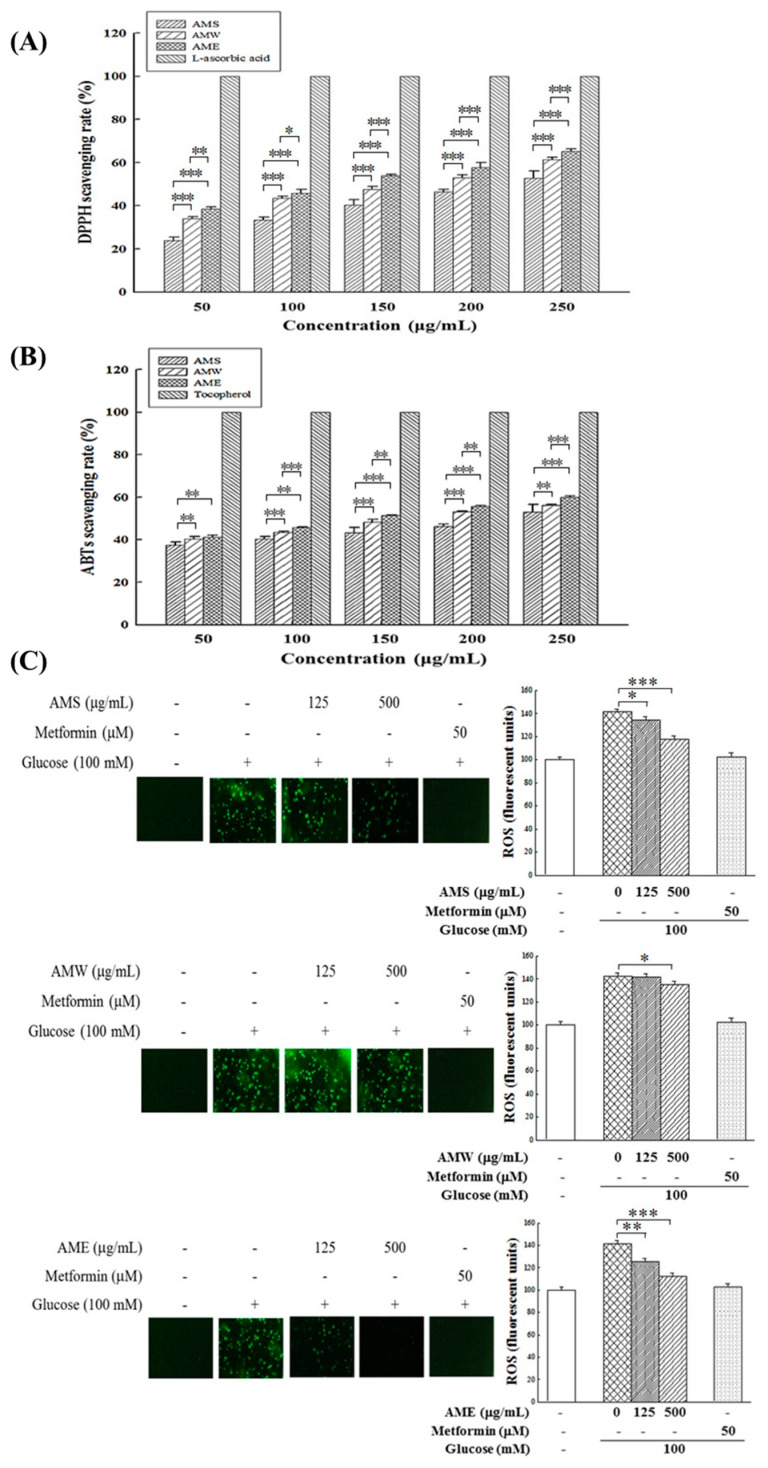
Effects of three AMf extracts on DPPH scavenging rate (**A**), ABTS scavenging rate (**B**), and intracellular ROS formation in β-cells under HG-stimulated conditions (**C**). Each set of data was derived from three and five separate replicate experiments, respectively. Statistical differences were determined using one-way ANOVA test. Levels of significance are indicated as * *p* < 0.05, ** *p* < 0.01, and *** *p* < 0.001. L-Ascorbic acid, α-tocopherol, and metformin were used as the positive control, respectively.

The antioxidant effects of AMf extracts were investigated by assessing their scavenging capacities on DPPH free radicals and ABTS free radicals, and ROS production in HG-stimulated RIN-m5f cells. As depicted in Figure 5A,B, the experimental results reveal that all three extracts exhibited notable DPPH and ABTS free radical scavenging abilities, and these effects were dose-dependent. The order of their activities was AME > AMW > AMS.

Regarding the influence of high glucose in medium on intracellular ROS production, results in Figure 5B indicate that exposure to 100 mM glucose stimulation resulted in a substantial increase in intracellular ROS production, by 141%. The intervention of the three extracts could reduce the production of intracellular ROS; among them, AME exhibited the most pronounced treatment effect, followed by AMS. AMW demonstrated only a marginal reduction effect. When the concentration was 500 µg/mL, the ROS production decreased to 112.1%, 119.6%, and 134.8%, respectively.

### 3.5. Effect on Antioxidant Enzymes in HG-Induced β-Cells

Based on earlier assessments, the performance of AME was superior to that of the other two extracts. Therefore, subsequent experiments were focused solely on AME.

**Figure 6:**
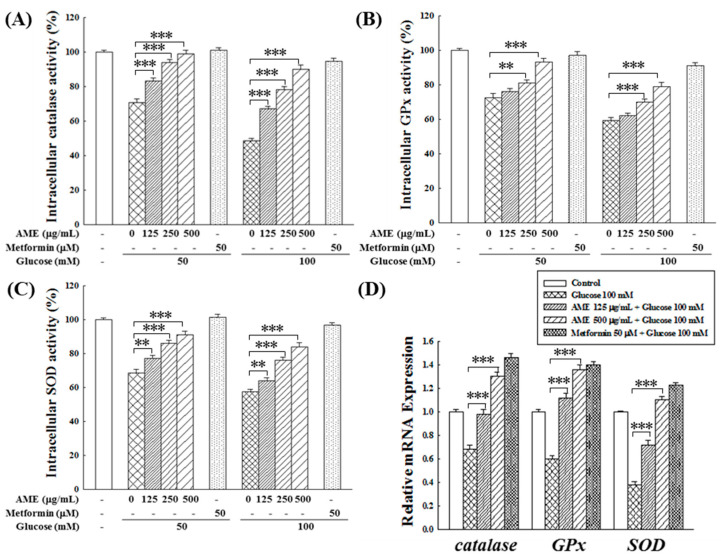
Effects of AME treatment on the intracellular activity of catalase (**A**), GPx (**B**), SOD (**C**), and the gene expression of these enzymes (**D**). Data were collected from five independent experiments. Statistical differences were determined using one-way ANOVA test. Levels of significance are indicated as ** *p* < 0.01, and *** *p* < 0.001.

Hyperglycemia is recognized for its potential to diminish the antioxidant system capacity in diabetic patients, resulting in elevated oxidative stress [26]. This study used β-cells as an experimental model to investigate whether AME possesses the capability to uphold the normal function of the intracellular antioxidant system against HG-induced oxidative stress. Figure 6A–C demonstrate a significant reduction in the activity of catalase (to 71.2% and 47.5%), GPx (to 70.3% and 58.2%), and SOD (to 69.7% and 55.0%) in environments with 50 and 100 mM glucose, respectively. The decline in enzyme activity was more pronounced with higher glucose concentrations. In such HG environments, adding the positive control, 50 μM metformin, could maintain the activities of the three antioxidant enzymes above 90%. Treatment with various concentrations of AME demonstrated a dose-dependent preservation of enzyme activities. Notably, the protective effect at a concentration of 500 μg/mL was comparable to that of the positive control.

The influence of AME on the gene expression of antioxidant enzymes is depicted in Figure 6D. Under the HG conditions of 100 mM glucose, the expression of *catalase*, *GPx*, and *SOD* genes in RIN-m5f cells significantly decreased to 67%, 61%, and 39%, respectively. Upon treatment with AME, there was a noticeable promotion in the expression of these three antioxidant enzyme genes. Specifically, when exposed to a concentration of 500 μg/mL, there was an increase in gene expression levels to 130.1%, 135.3%, and 110.2%, demonstrating a promotional effect comparable to that of the positive control.

### 3.6. Effect on Insulin Secretion in HG-Induced β-Cells

**Figure 7:**
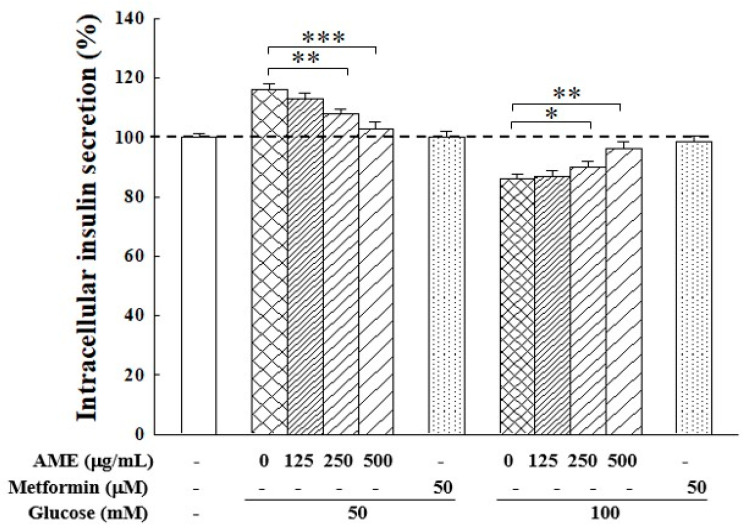
Influence of AME on insulin secretion in β-cells under HG induction. The dashed line is the auxiliary line for judgment and represents 100% secretion. Data were collected from five independent experiments. Statistical differences were determined using one-way ANOVA test. Levels of significance are indicated as * *p* < 0.05, ** *p* < 0.01, and *** *p* < 0.001.

The influence of the HG environment on insulin secretion in RIN-m5f cells is shown in Figure 7. Following a 24 h culture in medium with 50 mM and 100 M glucose, the intracellular insulin secretion rose to 115.1% when cells were with 50 mM glucose, but this dropped to 86.4% when cells were with 100 mM. This finding indicates there exists an imbalance in insulin secretion. Notably, treatment with AME demonstrated an effective regulation by reversing the HG effect, specifically decreasing insulin secretion in cells cultured with 50 mM glucose medium while increasing it in cells cultured with 100 mM glucose medium. The addition of 500 µg/mL AME resulted in insulin secretion levels of 103.3% and 95.8%, respectively. This finding suggests that AME exhibited a regulatory capability on insulin secretion in an HG environment.

### 3.7. Chemical Composition of AMf Extracts

**Figure 8:**
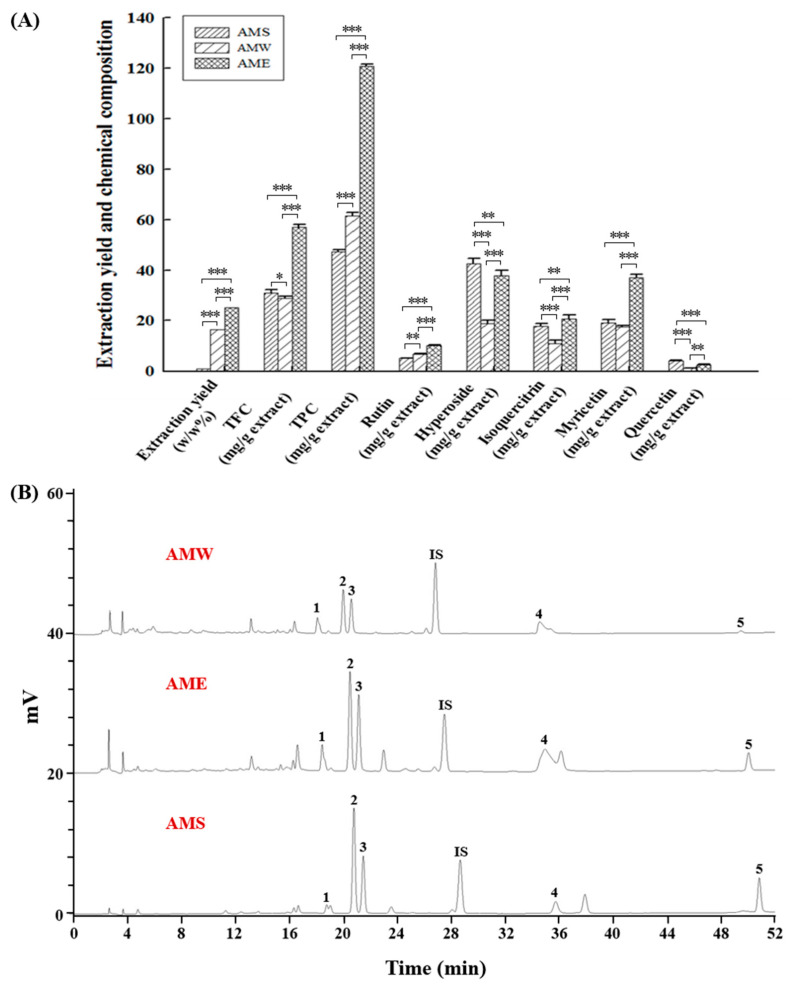
Quantitative measurement of the chemical constitution of the three AMf extracts. (**A**) Quantification of TFC, TPC, and individual components. (**B**) HPLC chromatogram depicts distinct peaks representing rutin (peak 1), hyperoside (peak 2), isoquercitrin (peak 3), myricetin (peak 4), quercetin (peak 5), and the internal standard naringin (peak IS). Compound identification and quantification were conducted using authentic standards (Sigma-Aldrich). Experimental data were obtained from three repeated experiments. Statistical differences were determined using one-way ANOVA test, with significance levels indicated as * *p* < 0.05, ** *p* < 0.01, *** *p* < 0.001.

Figure 8 depicts a comparative analysis of the three AMf extraction methods. The extraction rate of AME, where 95% ethanol was used to perform the extraction, was 25.2%. This exceeded the AMW extraction rate (16.4%), which used hot water for the extraction, by 1.54 times, and significantly surpassed the AMS extraction rate (1.0%) obtained through supercritical CO_2_ fluid extraction by 25.2 times. This underscores ethanol’s superior substance-extraction capability. Furthermore, among the three extracts, AME contained the highest TFC at 57.0 mg/g extract and TPC at 120.8 mg/g extract. In contrast, AMW had a higher TPC (61.5 mg/g extract), while AMS showed a greater TFC (30.9 mg/g extract). HPLC analysis revealed that hyperoside dominated the content among the three extracts, followed by myricetin, isoquercitrin, rutin, and quercetin. In AMS, hyperoside and quercetin content were the highest, whereas the other three chemical components were most abundant in AME.

## 4. Discussion

Huangkui capsule (HKC) has been utilized for the therapy of diabetic nephropathy and chronic kidney disease [17,18]. AME serves as the primary constituent of HKC, with its pharmacologically active components mainly comprising flavonoids. Nonetheless, to our understanding, there is still a scarcity of literature addressing the impact of AMf extracts on diabetes. Thus, our interest lies in exploring the protective effects of these extracts against the pathological mechanisms linked to diabetes.

Dysregulated carbohydrate and lipid metabolism are a common cause of type 2 diabetes, leading to elevated postprandial blood glucose levels and inadequate blood lipid control. When pancreatic α-amylase breaks down starch and intestinal α-glucosidase facilitates glucose absorption, blood glucose levels can abruptly spike, manifesting hyperglycemic symptoms in type 2 diabetes patients. Therefore, reducing dietary carbohydrate and lipid absorption through inhibiting α-amylase, α-glucosidase, and pancreatic lipase activities would be an effective strategy for managing diabetes symptoms. Furthermore, inhibiting α-glucosidase activity can mitigate carbohydrate end-product formation, thereby decreasing non-enzymatic protein glycation, glycated hemoglobin production, and collagen glycation end-product generation. As a result, this sort of intervention can improve the body’s biochemical parameters and delay or prevent the development of diabetic complications [27]. Numerous natural product extracts are recognized for their inhibitory ability on these enzymes [28].

The results presented in Figure 1 indicate that all three AMf extracts could significant inhibit α-glucoamylase and lipase activities; specifically, AME demonstrated the most pronounced inhibitory effect. However, all three AMf extracts exhibited an increasing effect on α-amylase activity, which involves breaking down starch into smaller molecules, subsequently hydrolyzing into glucose by α-glucoamylase and being absorbed in the intestine. As this effect may lead to an increase in small molecule carbohydrates derived from starch breakdown, it could potentially enhance the patient’s digestive system to avoid indigestion. Thus, the overall impact of AMf extracts on diabetic patients needs to be further evaluated.

The progression of diabetic complications can be accelerated by the formation of endogenous AGEs [6,7,8]. The data presented in Figure 2A,B of this study indicate that AMf extracts, more so AME, could significantly inhibit both Amadori product formation and dicarbonyl compound generation. Polyphenol and flavonoid compounds are regarded as potent glycation inhibitors [29,30]. In this study, we have shown AMf extracts contain substantial amounts of TPC and TFC. Therefore, AMf extracts are highly promising for further development as AGEs inhibitors, and may be considered as an alternative therapeutic strategy for preventing the occurrence and slowing the progression of diabetic complications [31,32].

Chronic hyperglycemia has been reported to promote glucose oxidation in mitochondria, leading to excessive formation of free radicals and increased oxidative stress in β-cells [33]. Normal cells possess intrinsic antioxidant defense mechanisms that regulate ROS levels and maintain the optimal redox state to sustain cellular homeostasis. However, when ROS production exceeds the capacity of these defenses, it can diminish the production and activity of antioxidant enzymes and exacerbate the oxidative stress. This oxidative imbalance can trigger apoptosis in β-cells, resulting in a progressive loss of β-cell function and population, inadequate insulin secretion, and ultimately an accelerated progression of diabetes. Hence, interventions targeting the mitigation of oxidative stress and apoptosis triggered by glucotoxicity, such as the use of antioxidants [34,35], are crucial therapeutic approaches to mitigate the risk of such pathological damage [36].

The results of this study indicate that the exposure of RIN-m5f cells to HG levels significantly reduced cell viability (Figure 3A). The treatment with AMf extract showed that all three extracts had minimal influence on RIN-m5f cell growth (Figure 3B–D). However, HG conditions with 50 mM or 100 mM glucose markedly inhibited RIN-m5f cell growth. Under these conditions, AME provided substantial protection, while AMS and AMW had limited protective effects (Figure 4).

Results in Figure 5A,B clearly show that all three AMf extracts exhibit significant in vitro and intracellular antioxidant activity, effectively reducing the levels of ROS stimulated by HG. Furthermore, AME can revert the HG-induced decrease in antioxidant enzyme activity and gene expression in β-cells (Figure 6).

The primary features of type 2 diabetes involve dysfunction in pancreatic β-cells and insulin resistance, both of which contribute to an imbalance in energy metabolism within the patient’s body. As blood glucose levels rise, pancreatic β-cells release insulin to regulate glucose levels in blood. Even worse, prolonged hyperglycemia-induced glucotoxicity can lead to β-cell death and dysfunction, and impede insulin production.

The data from Figure 7 illustrate that under 50 mM glucose conditions, insulin secretion in β-cells was significantly increased. On the other hand, when β-cells were cultured in 100 mM glucose conditions, insulin secretion was markedly decreased. This finding suggests that HG (50 mM glucose)-induced glucotoxicity has begun to up-regulate the β-cells to produce more insulin. Exposure to a high level of glucose (100 mM)-induced glucotoxicity has led to β-cell dysfunction and impeded insulin production. However, administration of AME could normalize insulin secretion modulation to regular levels. This demonstrates AME’s bidirectional regulatory effect on insulin secretion in β-cells responding differently to glucose concentration in medium. As shown in the results in Figure 4 and Figure 6, AME can also modulate the antioxidant system within β-cells and protect their viability. These factors should contribute to the maintenance of normal β-cell insulin secretion function.

As shown in Figure 8, the primary active constituents of AMf are polyphenols and flavonoids. There are five main compounds analyzed in this study which can serve as key indicators of AMf extracts [15,17]. These compounds, namely hyperoside [37,38], myricetin [39,40], isoquercitrin [41,42], rutin [43,44], and quercetin [45,46], have been reported to possess anti-diabetic properties or have potential in preventing diabetic complications.

In this study, we present the collective beneficial effects of AMf extracts on anti-diabetic outcomes. Across all tests, except for the enhancement of α-amylase activity, AME exhibited the highest activity, followed by AMS, whereas AMW showed no significant effect. The higher activities of AME as compared to AMS and AMW could be attributed mainly to its higher TPC and TFC (Figure 8A) and the greater content of its main constituent components (Figure 8B). It is also apparent that different extraction methods differed not only in yield but also in composition. This is because in natural product extraction methods, supercritical CO_2_ technology extracts moderately low polar components, water as a solvent favors highly polar components, and ethanol extraction can yield a wide range of polar components. Consequently, AME extract contains a higher content of phytochemicals.

## 5. Conclusions

AMf extracts exhibited potent inhibitory effects on α-glucosidase and lipase, along with significant suppression of AGE formation. Under HG conditions, AME exhibited promising intracellular antioxidative properties. It enhanced antioxidant enzyme activities and gene expression within RIN-m5f cells, and effectively reduced ROS generation, thereby preserving β-cell viability and maintaining the secretion of insulin at normal levels. The superior activity of AME among the three extracts might be attributed to its extraction method, which results in higher levels of TPC, TFC, and their key constituents.

As per our knowledge, this study is the first to delve into the utilization of AMf extracts for the purpose of preventing or managing diabetes. The outcomes outlined above provide a sound biochemical foundation for subsequent animal and clinical investigations on AME. To thoroughly understand the in vivo preventive effects and the underlying mechanisms, additional research is warranted.

## Figures and Tables

**Table 1:** Primer sequences utilized in the qRT-PCR assay.

Gene	Forward (5′−3′)	Reverse (5′−3′)
*Cat*	GCCATTGCCACAGGAAAGTA	CCTTGGTGAGATCGAATGGA
*GPx*	CCAAGCTCATCACCTGGTCT	TCGATGTCAATGGTCTGGAA
*SOD*	TGGCCGATGTGTCTATTGAA	CACCTTTGCCCAAGTCATCT
*GAPDH*	TGCACCACCAACTGCTTAGC	GGCATGGACTGTGGTCATGAG

## Data Availability

Data are contained within the article.
